# Supplementary material for: Genome-Wide Analysis of the TCP Transcription Factor Gene Family in Pepper (Capsicum annuum L.)
Source: Plants (Basel). 2024 Feb 26;13(5):641. doi: 10.3390/plants13050641 (PMC10934501; doi:10.3390/plants13050641)
Supplement: Supplementary file 1 [file plants-13-00641-s001.zip › Supplementary Figure S1.pdf]

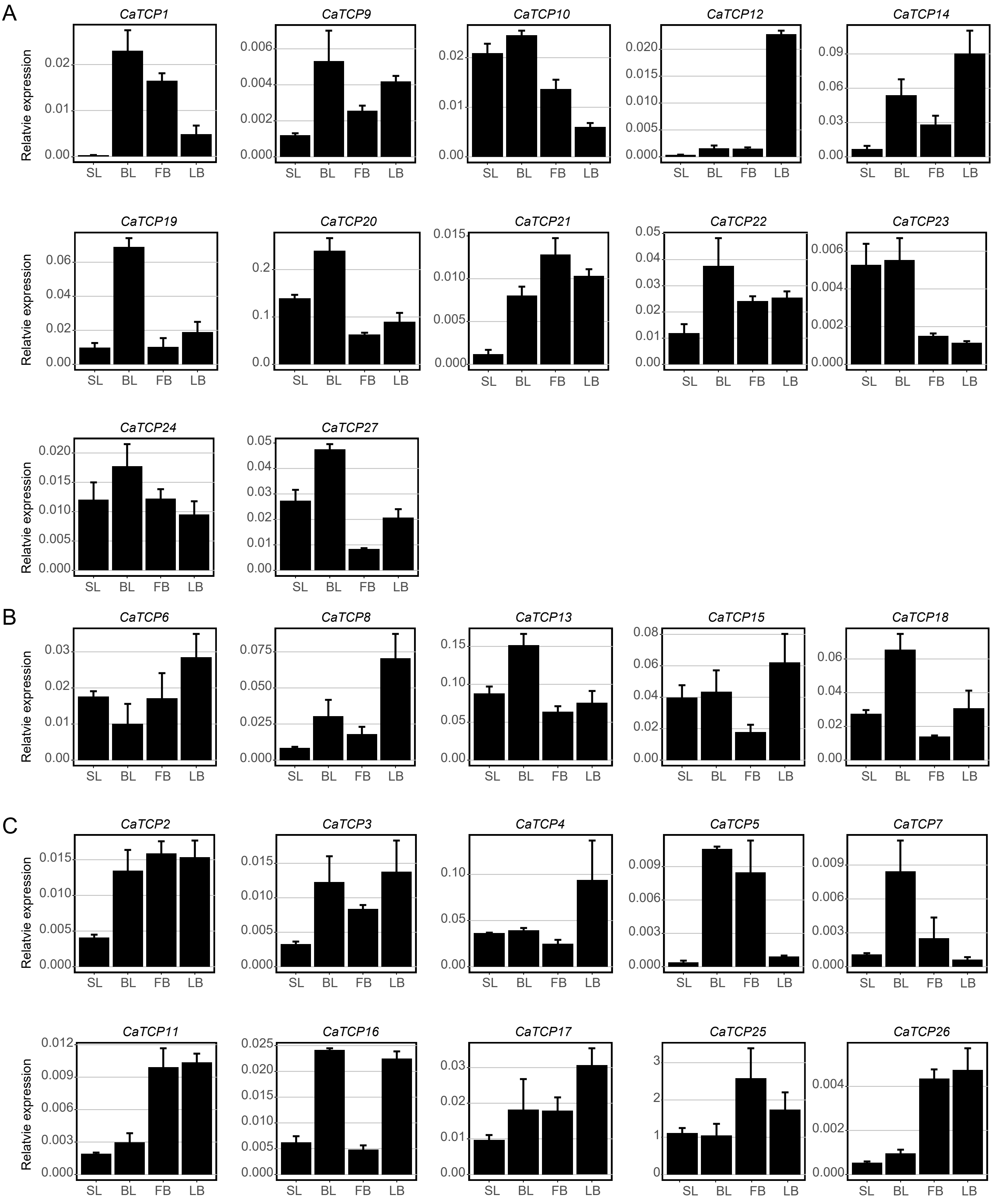

Figure S1 : Relative expression levels of 27 *CaTCPs* in stem leaf (SL), branch leaf (BL), flower bud (FB) and lateral bud (LB). **(A)** The expression levels of Class I *CaTCPs*, **(B)** subclass CYC/TB1 belonging to Class II, and **(C)** subclass CIN belonging to Class II. The value was mean of three replicates. SD was represented using error bar. Raw data were recorded in Table S8, 2−ΔΔCT method was used to calculate relative expression level.
