# Supplementary material for: Genome-Wide Analysis of the TCP Transcription Factor Gene Family in Pepper (Capsicum annuum L.)
Source: Plants (Basel). 2024 Feb 26;13(5):641. doi: 10.3390/plants13050641 (PMC10934501; doi:10.3390/plants13050641)
Supplement: Supplementary file 1 [file plants-13-00641-s001.zip › Supplementary Figure S2.pdf]

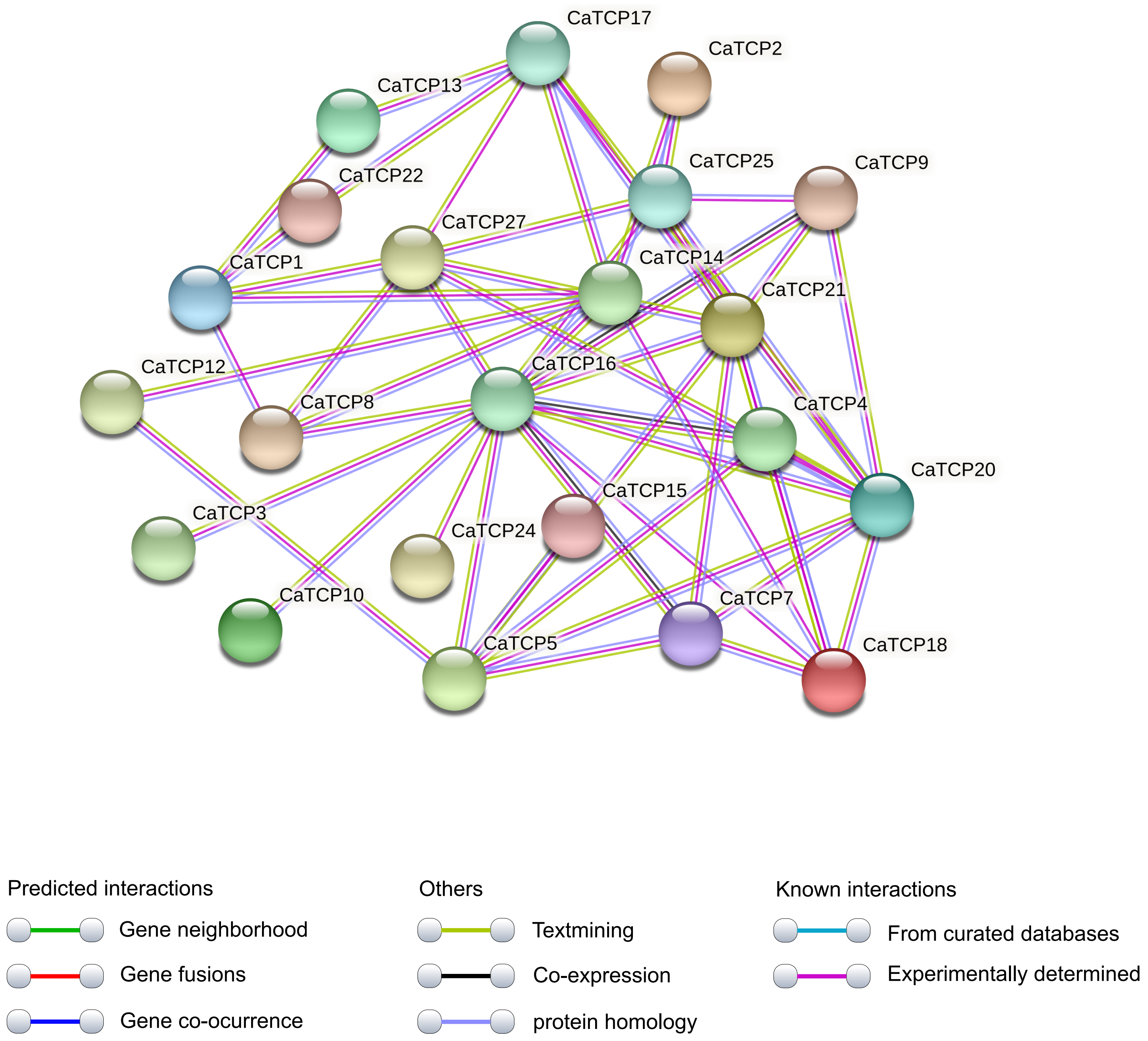

Figure S2: Predicted protein-protein interaction network of CaTCP proteins. Different data sources are remarked by various colors of the line. The String website was used to predict the protein-protein interaction network.
