# Supplementary material for: Genome-Wide Analysis of the TCP Transcription Factor Gene Family in Pepper (Capsicum annuum L.)
Source: Plants (Basel). 2024 Feb 26;13(5):641. doi: 10.3390/plants13050641 (PMC10934501; doi:10.3390/plants13050641)
Supplement: Supplementary file 1 [file plants-13-00641-s001.zip › Supplementary Figure S3.pdf]

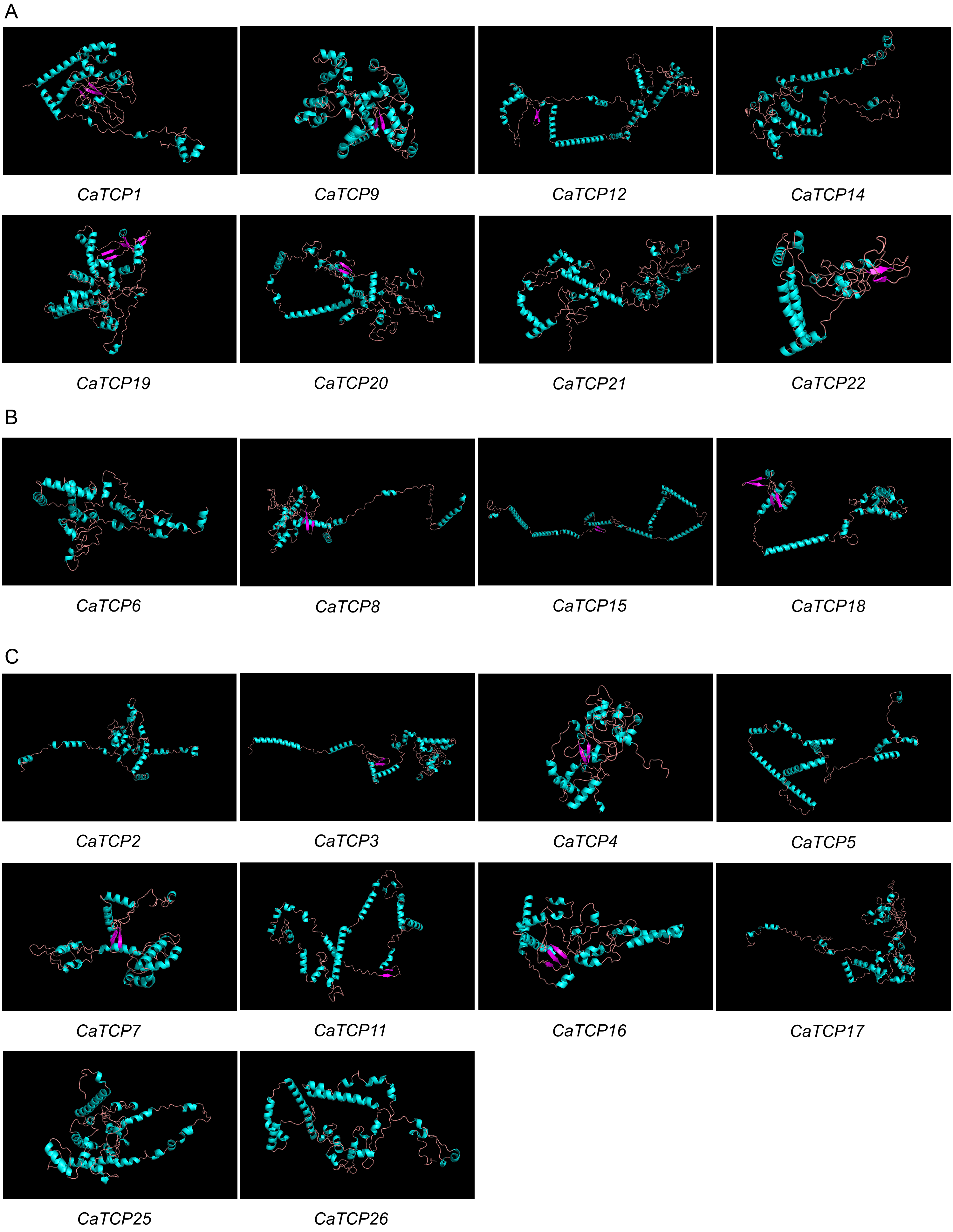

Figure S3: Predicted 3-dimensional models of CaTCP proteins, and α-helices, β-sheets, and random coils are remarked by cyan, red, and brown, respectively. **(A)** The Predicted 3-dimensional model of PCF subclass members. **(B)** The Predicted 3-dimensional model of CYC/TB1 subclass members. **(C)** The Predicted 3-dimensional model of CIN subclass members. AlphaFold2 website serve was used to predict the Three-Dimensional structure of CaTCP proteins.
